# Draft genome of *Fusarium solani* BAT10: an efficient polyurethane-degrading fungus isolated from a waste gas-treating biofilter

**DOI:** 10.1128/mra.01148-25

**Published:** 2026-09-11

**Authors:** Jiří Bárta, Jiří Mach, Martin Halecký

**Affiliations:** 1 Department of Ecosystem Biology, Centre for Polar Ecology, Faculty of Science, University of South Bohemia in Ceske Budejovice 204738, České Budějovice, Czechia; 2 Department of Biotechnology, Faculty of Food and Biochemical Technology, University of Chemistry in Prague 52735 , Prague, Czechia; University of California Riverside, Riverside, California, USA

**Keywords:** *Fusarium*, plastic, biodegradation

## Abstract

Plastic degradation by microorganisms offers a natural mechanism for the breakdown of persistent pollutants. Here, we report the draft genome sequence of *Fusarium solani* strain BAT10, isolated from a waste gas-treating biofilter and capable of degrading aliphatic polyurethane foams as the only source of carbon, nitrogen, and energy.

## ANNOUNCEMENT

The ability of microorganisms including fungi (1, 2) to degrade various plastic polymers has been extensively studied for over a decade (3). The *Fusarium solani* strain BAT10 was isolated from a mixed culture of a biofilter long-term treating waste gases with a mixture of acetone and styrene (Czech Republic, Prague; 50°6′12.104″N, 14°23′23.568″E) using Rose-Bengal Chloramphenicol Agar Base CM 549 with a selective supplement SR 78 selective agar (Oxoid, England) at a temperature of bioreactor operation of 20°C (4). Efficient degradation of both acetone and styrene, and all their detected degradation products, was observed (4). Fungus was previously used as a model fungal degrader of polyurethanes, namely pure aliphatic polyurethane foams from a trimer of hexamethylene diisocyanate and poly(diethylene glycol adipate) diol (5–7).

*F. solani* mycelium taken from Sabouraud 4% Glucose Agar with chloramphenicol (Carl ROTH, Germany) was used as inoculum for 2-day suspension cultivation of biomass acquisition for DNA extraction. The cultivation conditions were as follows: 1 L Erlenmeyer flask, rotations per minute (RPM) of 200, temperature of 25°C, and basic salt medium with glucose (5 g/L). Following centrifugation (10 min at 10,000 rpm), approximately 5 g of fungal biomass was aseptically transferred to a precooled sterile mortar. The biomass was frozen with liquid nitrogen and ground to a fine powder using a sterile pestle. Approximately 2.5 g was transferred to extraction buffer, and DNA was extracted following the protocol of DNeasy PowerLyzer Microbial Kit (Qiagen, USA). The quality of DNA was confirmed by agarose gel electrophoresis, and the concentration was determined fluorometrically by Qubit (ThermoFisher Scientific, UK). The purified DNA extract was sent to SEQme company for library preparation and whole-genome sequencing. Library preparation was done using NEBNext Ultra II DNA Library Prep Kit for Illumina and NEBNext Multiplex Oligos for Illumina (Unique Dual Index Primer Pairs). Library QC was confirmed by Agilent Bioanalyzer 2100 High Sensitivity DNA Kit, Invitrogen Collibri Library Quantification Kit, and Qubit 1X dsDNA High-Sensitivity Assay Kit. The sequencing platform was NovaSeq X Plus, 2 × 150 bp. Raw fastq reads were *de novo* assembled by SPAdes v3.15.3 (8) with default parameters. The genome size of *F. solani* strain BAT10 was estimated to be 51,526,594 bp with 51.34% GC (Table 1). The completeness of the genome was determined by BUSCO v. 5.8.0. In total, 758 BUSCO groups were searched, of which 753 were identified as complete, corresponding to 99.3% completeness. The majority of complete BUSCOs were present as single-copy genes, with 747 BUSCOs classified as complete and single copy (98.5%), while only six BUSCOs were detected as duplicated (0.8%). One BUSCO was classified as fragmented (0.1%), and four BUSCOs were missing (0.5%). Among the complete BUSCOs, 38 contained internal stop codons.

**TABLE 1:** QUAST summary of *F. solani* strain BAT10 genome assembly and annotation^*a*^

Parameter	Genome assembly
Contigs	
Contigs (≥0 bp)	485
Contigs (≥1,000 bp)	345
Contigs (≥10,000 bp)	201
Contigs (≥100,000 bp)	94
Contigs (≥1,000,000 bp)	9
Largest contig	3,298,583
Total length	
Total length (≥0 bp)	51,526,594
Total length (≥1,000 bp)	51,434,554
Total length (≥10,000 bp)	50,891,223
Total length (≥100,000 bp)	47,263,454
Total length (≥1,000,000 bp)	15,531,990
N50	653,004
N75	308,289
L50	21
L75	50
GC (%) Mean coverage (unweighted)	51.34 337.8

^*a*^ All statistics are based on contigs of size ≥500 bp, unless otherwise noted (e.g., “Contigs (≥0 bp)” and “Total length (≥0 bp)” include all contigs).

We also checked for the presence of mitochondrial contigs in assembly. We identified contig NODE_129_length_38732_cov_8394.574389 with exceptionally low AT content and high coverage of 8,394. We searched the available proteins of all *Fusarium* species in GenBank using the BLASTX algorithm. We confirmed the presence of mitochondrial ATP synthase and cytochromes, which confirmed that this contig is potentially mitochondrial.

## Data Availability

The raw reads were deposited in the Sequence Read Archive (SRA) under the Bioproject ID PRJNA1327974, BioSample ID SAMN51303692. This Whole Genome Shotgun project has been deposited at DDBJ/ENA/GenBank under the accession JBRIIE000000000. The version described in this paper is version JBRIIE010000000.
